# IL1RAP Is Associated With an Inflammation–Immunity–Related State in Skin Cutaneous Melanoma: Integrative Evidence From Pan‐Cancer Data and Melanoma Immunotherapy Cohorts

**DOI:** 10.1155/ijog/3751729

**Published:** 2026-09-01

**Authors:** Chenxi Jiang, Hairui Li, Jianqiong Huang, Maojun Chen

**Affiliations:** ^1^ Department of Plastic and Aesthetic Surgery, West China Tianfu Hospital, Sichuan University, Chengdu, China, scu.edu.cn; ^2^ Department of Plastic and Aesthetic Surgery, West China Hospital, Sichuan University, Chengdu, China, scu.edu.cn; ^3^ Department of Neurosurgery, West China Hospital, Sichuan University, Chengdu, China, scu.edu.cn

**Keywords:** cancer inflammation, IL1RAP, immune microenvironment, immunotherapy, integrative bioinformatics, pan-cancer analysis, skin cutaneous melanoma, tumor immunity

## Abstract

**Background:**

The crosstalk between inflammation and immunity plays a central role in tumor progression, immune evasion, and therapeutic response. Interleukin‐1 receptor accessory protein (IL1RAP) is a key adaptor in inflammatory signaling, yet its immunological relevance and clinical implications in skin cutaneous melanoma (SKCM) remain largely unexplored.

**Methods:**

We performed an integrative analysis combining pan‐cancer and melanoma‐focused datasets. Bulk transcriptomic, single‐cell, spatial transcriptomic, genomic alteration, pharmacogenomic, and clinical survival data were obtained from TCGA, GTEx, GEO, ENA, and other public resources. IL1RAP expression was evaluated across cancer types in relation to diagnostic performance, immune subtypes, survival outcomes, functional pathway activity, immune–genomic states, somatic alterations, and drug‐response metrics. Melanoma‐focused analyses examined immune infiltration, methylation‐derived tumor‐infiltrating lymphocyte (MeTIL) scores, and exploratory survival associations in five treatment cohorts; the survival groups were defined using cohort‐specific optimal cutoffs rather than median splits.

**Results:**

IL1RAP expression differed between tumor and normal tissues in multiple cancers, although the direction and magnitude varied by cancer type. Pan‐cancer survival associations were likewise context dependent. Single‐cell and spatial transcriptomic resources indicated cell‐type and spatial heterogeneity of IL1RAP expression within tumor microenvironments. Pathway, immune–genomic, and pharmacogenomic analyses identified exploratory associations with functional states, genomic features, and drug‐response metrics. In SKCM, IL1RAP expression was associated with several immune‐infiltration estimates and higher MeTIL scores. Across five melanoma immunotherapy cohorts, the direction and magnitude of the overall survival associations varied substantially.

**Conclusions:**

This retrospective integrative analysis suggests that IL1RAP may mark an inflammation–immunity–related state in SKCM. The heterogeneous associations across cancers and melanoma treatment cohorts support further validation but do not establish IL1RAP as a causal regulator, a treatment‐response predictor, or a therapeutic target.

## 1. Introduction

Skin cutaneous melanoma (SKCM) is an immunogenic malignancy in which the tumor microenvironment (TME) and inflammatory signaling jointly shape tumor evolution and treatment response [1, 2]. Beyond tumor‐intrinsic oncogenic programs, melanoma behavior is strongly conditioned by the quality, location, and functional state of immune infiltration, as well as by inflammatory mediators that govern antigen presentation, myeloid remodeling, T‐cell priming, and immune suppression [3]. This inflammation–immunity interplay is particularly relevant in melanoma because immune checkpoint blockade has become a major therapeutic modality, yet only a subset of patients derive durable benefit, highlighting the need for biomarkers that reflect the inflammatory–immune context and predict clinically meaningful outcomes [4].

Inflammatory signaling is not a uniform “pro‐” or “anti‐” tumor phenomenon; rather, it can promote tumor control or immune escape depending on cellular sources, pathway wiring, and spatial organization within the TME [5]. A central challenge is that many inflammation‐associated molecules exert pleiotropic effects across cell types, and bulk tumor measurements often conflate malignant, immune, and stromal contributions [6]. As a result, biomarkers that are framed purely as “inflammatory genes” without microenvironmental localization and cross‐platform validation may have limited interpretability or translational value [7]. An integrative strategy—linking bulk transcriptomics to single‐cell and spatial evidence, genomic context, and therapeutic response—can help distinguish context‐dependent associations from reproducible, clinically informative patterns [8].

Interleukin‐1 receptor accessory protein (IL1RAP) is a membrane‐associated coreceptor required for signal transduction by several interleukin‐1 family pathways, positioning it at a biologically plausible intersection between inflammation and immunity [9]. IL1RAP was therefore investigated as an a priori, literature‐informed candidate on the basis of its central signaling role, cell‐surface localization, and reported relevance in malignant and inflammatory settings [9–11]. This study was not designed as an unbiased family‐wide screen of interleukins or interleukin receptors; accordingly, the analyses should be interpreted as a targeted characterization of IL1RAP rather than as evidence that it is the uniquely prioritized member of this gene family. Melanoma‐focused evidence remains limited, motivating evaluation of whether IL1RAP expression is associated with immune contexture and clinical outcomes in this disease.

To address this question, we performed a systematic, multilayered analysis that uses pan‐cancer data as a contextual framework while anchoring interpretation in SKCM [12]. We profiled IL1RAP across cancers with respect to tumor–normal expression patterns, diagnostic performance, immune subtype distributions, and survival associations. We then integrated single‐cell and spatial transcriptomic resources to examine the cellular and spatial distribution of IL1RAP and used pathway‐level analyses to characterize biological programs associated with its expression. In parallel, we assessed genomic context through copy‐number and somatic‐mutation landscapes and explored pharmacogenomic correlations across multiple drug‐response resources.

Because immune‐related associations were observed in SKCM and melanoma‐specific characterization was limited, we further examined the relationship between IL1RAP and immune infiltration using complementary deconvolution strategies. We additionally evaluated MeTIL scores and exploratory survival associations in five publicly available melanoma immunotherapy cohorts. Together, these analyses evaluate whether IL1RAP may serve as an inflammation–immunity–associated state marker in melanoma, without assuming a causal or predictive role.

## 2. Methods

### 2.1. Data Sources, Patient Cohorts, and Preprocessing

Transcriptomic expression data for pan‐cancer analyses were obtained from The Cancer Genome Atlas (TCGA) and the Genotype‐Tissue Expression (GTEx) project via the UCSC Xena platform, including the tcga_RSEM_gene_tpm and gtex_RSEM_gene_tpm datasets [13–15]. Only primary tumor samples from TCGA were included, and normal tissue samples were matched to corresponding tumor types based on anatomical origin.

Additional multiomics data, including somatic single‐nucleotide variants (SNVs), copy‐number variation (CNV) profiles, DNA methylation data, and corresponding clinical survival information, were retrieved from TCGA for integrative pan‐cancer analyses. Single‐cell RNA sequencing data were obtained from the Tumor Immune Single‐cell Hub (TISCH) database, including multiple pan‐cancer TME datasets [16]. Spatial transcriptomic datasets based on the 10x Genomics Visium platform were obtained from publicly available spatial transcriptomics resources [17].

Pharmacogenomic drug‐response data were collected from four large‐scale resources: Genomics of Drug Sensitivity in Cancer (GDSC1 and GDSC2) [18], Cancer Therapeutics Response Portal (CTRP) [19], and PRISM [20]. For SKCM‐focused analyses, bulk RNA sequencing, DNA methylation, and clinical data from TCGA‐SKCM were used for immune infiltration and MeTIL analyses. Five publicly available melanoma immunotherapy datasets were used for exploratory survival analyses: GSE78220 (28 expression profiles; anti‐PD‐1 therapy) [21], GSE91061 (109 expression profiles; anti‐PD‐1 therapy) [22], GSE106128 (47 longitudinal blood expression profiles; dendritic cell–based vaccination), Nathanson_2017 (24 records; CTLA‐4 blockade), and PRJEB23709 (91 records; anti‐PD‐1 alone or combined anti‐PD‐1/anti‐CTLA‐4 therapy) [23]. These resources differed in treatment modality, sampling time, unit of analysis, and source processing; therefore, each cohort was analyzed separately and was not pooled with the others.

For TCGA–GTEx comparisons, transcriptomic expression values were converted to transcripts per million (TPM) and log2‐transformed where appropriate [24]. TPM values were standardized within each cancer type using *Z*‐score transformation ((*x* − *μ*)/*σ*). Samples with *Z*‐scores < −3 or > 3 were treated as outliers and removed, and cancer types with fewer than three normal samples after filtering were excluded. The five melanoma treatment cohorts were not merged, and no cross‐cohort batch correction was applied because their platforms, biospecimens, sampling schedules, and treatment contexts were not directly comparable. Analyses were performed within each cohort, which limits cross‐cohort effect‐size comparability.

### 2.2. Pan‐Cancer Expression, Diagnostic, Immune Subtype, and Survival Analyses

Differential expression of IL1RAP between tumor and normal tissues across cancer types was assessed using the Wilcoxon rank‐sum test based on *Z*‐score–normalized expression values. Receiver operating characteristic (ROC) curve analyses were conducted to evaluate the diagnostic performance of IL1RAP expression in distinguishing tumor tissues from normal tissues, using both the TCGA‐only cohort and the combined TCGA–GTEx cohort. Area under the ROC curve (AUC) values and corresponding 95% confidence intervals (CIs) were calculated.

Immune subtype annotations (C1–C6) were obtained from the TCGA immune landscape framework [25]. Tumor samples were stratified into high‐ and low‐expression groups based on the median IL1RAP expression level within each cancer type. Differences in immune subtype distributions between groups were evaluated using the chi‐square test.

Univariate Cox proportional‐hazards regression analysis was performed to assess the association between IL1RAP expression and survival outcomes across cancers, including overall survival (OS), disease‐specific survival (DSS), disease‐free interval (DFI), and progression‐free interval (PFI). Hazard ratios (HRs) and 95% CIs were estimated using the survival R package.

### 2.3. Single‐Cell and Spatial Transcriptomic Analyses

Pan‐cancer single‐cell RNA sequencing data were obtained from the TISCH database, and IL1RAP expression was summarized at the cell‐type level. *Z*‐score normalization was applied to standardized expression values. Euclidean distance matrices were calculated to evaluate similarity across cell populations, and hierarchical clustering was performed using Ward′s linkage method to optimize heatmap visualization.

Spatial transcriptomic analyses were conducted using pan‐cancer 10x Genomics Visium datasets [26]. Cell‐type deconvolution of spatial spots was performed based on reference single‐cell RNA sequencing profiles using the Cottrazm framework, including tumor boundary definition, spatial deconvolution, and subspot‐level spatial reconstruction. Spatial regions were annotated according to the dominant cell type within each spot. Average IL1RAP expression levels were calculated for each annotated region and standardized using *Z*‐score transformation. Heatmaps were generated using the pheatmap package.

### 2.4. Functional Enrichment and Pathway Activity Analyses

Gene set enrichment analysis (GSEA) was performed using the clusterProfiler R package [27] to evaluate pathway‐level differences associated with IL1RAP expression across cancer types. For each tumor type, samples in the top 30% and bottom 30% of IL1RAP expression were defined as high‐ and low‐expression groups, respectively. This contrast‐based threshold was chosen to increase expression separation while retaining adequate sample numbers; it was used only for GSEA and not for Figure 1 survival analyses. Differential expression analysis between groups was conducted using the limma package [28] to obtain log2 fold‐change values. Genes were ranked by log2 fold change and subjected to GSEA using Hallmark and KEGG metabolism–related gene sets. Enrichment scores (ES), normalized enrichment scores (NES), and Benjamini–Hochberg false discovery rate (FDR)–adjusted *p* values were calculated.

Figure 1Association of IL1RAP with MeTIL scores and overall survival in melanoma immunotherapy datasets. (A) MeTIL‐score distributions in median‐defined IL1RAP‐high and IL1RAP‐low TCGA‐SKCM groups; boxes show the median and interquartile range, and the two‐sided Wilcoxon rank‐sum test gave *p* = 0.015. (B–F) OS Kaplan–Meier curves using cohort‐specific optimal IL1RAP cutoffs selected by the minimum two‐sided log‐rank *p* value, not median cutoffs: (B) GSE78220, anti‐PD‐1, high/low *n* = 25/2, HR = 0.088 (95% CI 0.014–0.540), *p* = 0.0083; (C) GSE91061, anti‐PD‐1, *n* = 91/10, HR = 10.662 (1.476–77.037), *p* = 0.019; (D) GSE106128, dendritic cell–based vaccination with longitudinal expression profiles, *n* = 27/20, HR = 3.150 (1.415–7.012), *p* = 0.00497; (E) Nathanson_2017, CTLA‐4 blockade, *n* = 20/4, HR = 0.199 (0.057–0.697), *p* = 0.0116; and (F) PRJEB23709, anti‐PD‐1 alone or combined anti‐PD‐1/anti‐CTLA‐4, *n* = 80/11, HR = 8.021 (1.085–59.295), *p* = 0.0414. Red denotes the high‐expression group and blue the low‐expression group. HRs are from univariate Cox models. These data‐derived cutoffs and heterogeneous treatment/sampling contexts make the analyses exploratory rather than confirmatory or directly comparable across cohorts.

**Figure:**
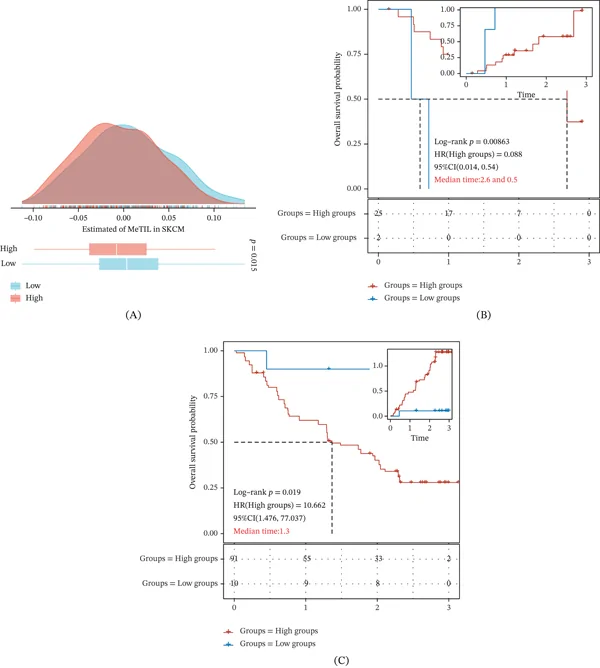
(A)

**Figure:**
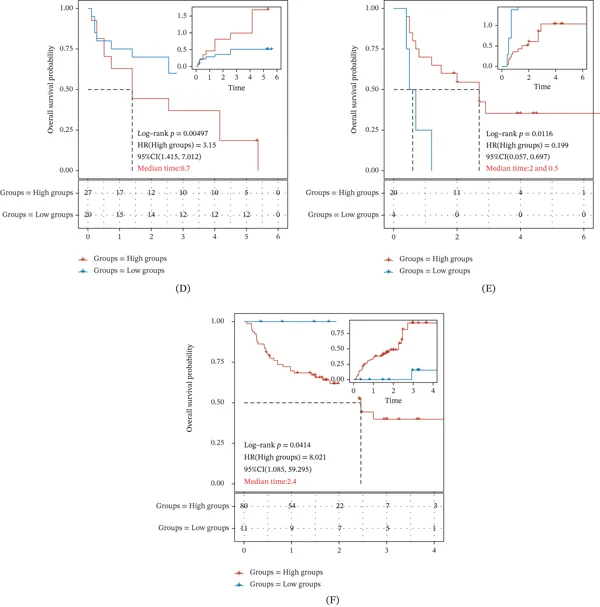
(B)

Functional state association analyses were conducted using 14 cancer cell functional state gene sets obtained from the CancerSEA database [29]. Pathway activity scores were calculated using the GSVA package [30] with the *Z*‐score method, followed by standardization using the scale function. Pearson correlation analysis was performed to assess associations between IL1RAP expression levels and functional state scores.

### 2.5. Genomic Alteration Analyses

Patients were stratified into four groups (Q1–Q4) according to quartiles of IL1RAP expression. Immune‐related and genomic instability–related scores were summarized for each cancer type, following the immune response and genomic state framework described by Thorsson et al. [25], and intragroup mean values were calculated with missing values ignored. Heatmaps were generated using the pheatmap package with row‐wise standardization.

Differences in IL1RAP expression across CNV categories were assessed using the Kruskal–Wallis rank‐sum test. Somatic SNV data across 33 cancer types were analyzed to characterize mutation patterns, with noncoding and silent variants excluded from frequency calculations. Mutation frequencies were calculated as the proportion of mutated samples within each cancer type, and oncoplots and mutation waterfall plots were generated using the maftools package [31]. Spearman correlation analysis was performed to evaluate associations between IL1RAP expression and multiple gene set–based functional scores across cancers, with correlation coefficients and *p* values visualized using radar plots.

### 2.6. Drug Sensitivity Analyses

Spearman correlation analysis was used to assess associations between IL1RAP expression and drug‐response profiles across pan‐cancer pharmacogenomic resources. Drug‐response metrics included area under the dose–response curve (AUC) values from CTRP and PRISM and half‐maximal inhibitory concentration (IC50) values from GDSC1 and GDSC2. For both types of metrics, a negative correlation indicated that higher IL1RAP expression was associated with lower AUC or IC50 (greater apparent sensitivity), whereas a positive correlation indicated higher AUC or IC50 (lower apparent sensitivity). Drugs were ranked by nominal statistical significance, and top‐ranked compounds were visualized. These correlations were treated as exploratory and were not interpreted as evidence that IL1RAP directly mediates drug sensitivity.

### 2.7. Immune Infiltration, MeTIL, and Immunotherapy Analyses in SKCM

**Figure 2:**
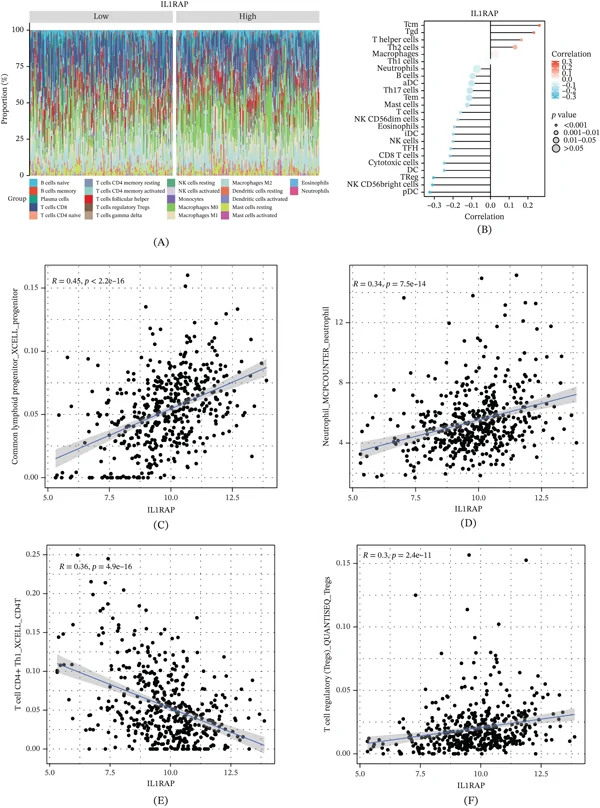
Associations between IL1RAP expression and immune‐infiltration estimates in TCGA‐SKCM. (A) CIBERSORT LM22 proportions in median‐defined IL1RAP‐high and IL1RAP‐low groups; available source‐workflow estimates were displayed without an additional manuscript‐stage deconvolution *p* filter. Colors identify the 22 cell types. (B) Spearman correlations between continuous IL1RAP expression and ssGSEA scores from 24 immune‐cell signatures. Color shows correlation direction and magnitude, whereas point size indicates the nominal *p* value category. (C–F) Representative continuous‐expression correlations from xCell, MCP‐counter, and QUANTISEQ; *R*, two‐sided nominal *p* values, fitted lines, and 95% confidence bands are shown. Positive *R* indicates a concordant association and negative *R* an inverse association. No global FDR correction was applied.

Immune cell infiltration in TCGA‐SKCM samples was estimated using complementary approaches. Relative proportions of 22 immune cell types were obtained with CIBERSORT using the LM22 signature matrix [32]. Samples were divided at the median IL1RAP expression for the descriptive high‐ versus low‐expression composition plot. The available source‐workflow CIBERSORT estimates were retained, and no additional manuscript‐stage filtering by the CIBERSORT deconvolution *p* value was applied. Immune‐infiltration scores were also calculated by single‐sample gene set enrichment analysis (ssGSEA), implemented in the GSVA package using 24 curated immune‐cell marker sets. Spearman correlations were used to evaluate associations between IL1RAP and immune‐infiltration estimates. The seven‐method framework comprised TIMER, CIBERSORT, CIBERSORT‐ABS, QUANTISEQ, MCP‐counter, xCell, and EPIC; representative xCell, MCP‐counter, and QUANTISEQ results are displayed in Figure 2C–F. MeTIL scores were calculated from the five published CpG markers cg20792833, cg23642747, cg12069309, cg20425130, and cg21554552 using the normalized PCA parameters reported by Jeschke et al. [33]. The five probe beta values were centered and scaled using the published discovery‐cohort parameters, and the first principal‐component projection was used as the MeTIL score. Samples lacking any required probe value or a derived MeTIL score were omitted; no additional probe‐level imputation was performed. The resulting scores were *Z*‐standardized for visualization. TCGA‐SKCM samples were divided at the median IL1RAP expression, and MeTIL‐score distributions were compared with the Wilcoxon rank‐sum test [33, 34].

For exploratory survival analyses, the five melanoma immunotherapy datasets were analyzed separately. Within each dataset, an optimal IL1RAP expression cutoff was selected by evaluating candidate cut points and choosing the threshold that produced the smallest two‐sided log‐rank *p* value. Thus, Figure 1B–F does not use cohort‐specific medians, and the high‐ and low‐expression group sizes differ markedly among cohorts. Kaplan–Meier curves, log‐rank tests, and univariate Cox models were used to summarize OS associations. Because these cutoffs were selected from the same data used for outcome testing, the resulting *p* values and HRs may be optimistic and should be considered exploratory. No pooled model or formal response‐category analysis was performed.

### 2.8. Statistical Analysis

All statistical analyses were performed using R software (Version 4.2.1). Continuous variables were compared between two groups using the Wilcoxon rank‐sum test, and comparisons among multiple groups used the Kruskal–Wallis test where appropriate. Correlations were assessed using Spearman or Pearson methods according to analytical context. Survival analyses used Kaplan–Meier curves and two‐sided log‐rank tests; HRs and 95% CIs were estimated with univariate Cox proportional‐hazards models, and the proportional‐hazards assumption was evaluated using Schoenfeld residuals. Diagnostic performance was summarized using ROC curves and AUC values with 95% CIs. Benjamini–Hochberg correction was applied to GSEA results, and FDR‐adjusted *p* values are reported for that analysis. Unless explicitly labeled as FDR‐adjusted, *p* values in the pan‐cancer correlations, survival screens, immune‐infiltration analyses, genomic associations, and pharmacogenomic analyses are nominal and were not retrospectively corrected; these analyses are interpreted as exploratory. All tests were two‐sided, and nominal *p* < 0.05 was used as a screening threshold where applicable.

## 3. Results

### 3.1. Pan‐Cancer Expression Pattern, Diagnostic Performance, and Immune Subtype Association of IL1RAP

**Figure 3:**
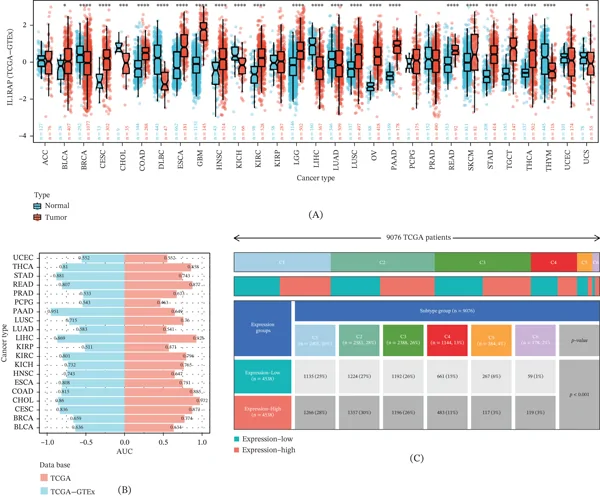
Pan‐cancer expression pattern, diagnostic performance, and immune‐subtype association of IL1RAP. (A) IL1RAP expression in tumor and normal tissues using TCGA paired tumor–normal, TCGA tumor versus TCGA normal, and TCGA tumor versus GTEx normal comparisons. Available samples after the filtering described in Section 2 were included; *n* therefore varies by cancer type. Two‐group comparisons used two‐sided Wilcoxon rank‐sum tests, and displayed *p* values are nominal. (B) ROC analysis of tumor–normal discrimination in TCGA‐only and TCGA–GTEx datasets; AUC and 95% CI are shown. (C) TCGA immune‐subtype distributions in median‐defined IL1RAP‐high and IL1RAP‐low groups; the two‐sided chi‐square test was used (*p* < 0.001). Colors identify cancer types or immune subtypes as labeled in the panels.

Pan‐cancer differential expression analysis showed that IL1RAP was dysregulated across multiple cancer types, with tumor tissues exhibiting higher expression than corresponding normal tissues in a substantial proportion of cancers, although the direction and magnitude of the difference varied by tumor context (Figure 3A). ROC analyses further indicated that IL1RAP had variable but generally informative diagnostic performance across selected cancer types in both the TCGA‐only and TCGA–GTEx settings, with particularly strong discrimination observed in cancers such as CHOL, READ, LIHC, and THCA, whereas the relative performance of the two datasets differed depending on cancer type rather than showing a uniformly improved pattern in the combined cohort (Figure 3B). Analysis of TCGA immune subtypes revealed a significant difference in subtype distribution between the IL1RAP high‐ and low‐expression groups (*p* < 0.001) (Figure 3C). Specifically, the high‐expression group showed higher proportions of C1 and C2 subtypes, whereas the low‐expression group contained relatively more C4 and C5 cases, although the proportion of C3 was largely comparable between the two groups. These findings indicate that IL1RAP is broadly altered across cancers and is associated with intertumoral immune heterogeneity at the pan‐cancer level.

### 3.2. Pan‐Cancer Prognostic Relevance of IL1RAP Across Multiple Survival Endpoints

**Figure 4:**
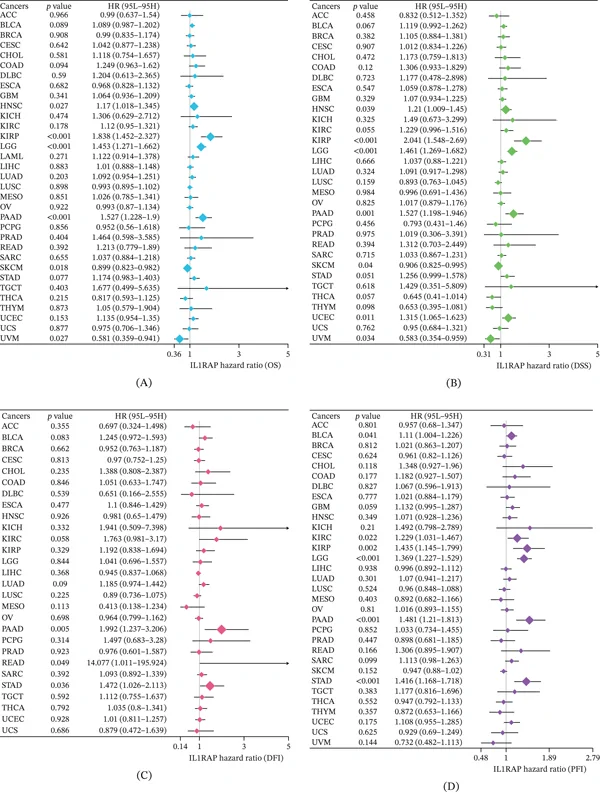
Pan‐cancer survival associations of IL1RAP in continuous‐expression univariate Cox models. Forest plots show HRs and 95% CIs for (A) overall survival, (B) disease‐specific survival, (C) disease‐free interval, and (D) progression‐free interval. Available cases with the relevant endpoint were included, so *n* varies by cancer type and endpoint. HR > 1 indicates greater estimated hazard per unit increase in IL1RAP expression; HR < 1 indicates lower estimated hazard. Displayed *p* values are two‐sided and nominal; no pan‐cancer FDR correction was applied.

Univariate Cox regression identified associations between IL1RAP expression and survival outcomes in multiple cancer types, but both direction and magnitude varied by tumor context (Figure 4A–D). For OS, higher IL1RAP expression was associated with greater estimated risk in several malignancies, whereas inverse associations were observed in others (Figure 4A). Similar cancer‐dependent patterns were observed for DSS, DFI, and PFI (Figure 4B–D). The forest plots summarize continuous‐expression Cox models; HR > 1 indicates greater estimated risk per unit increase in IL1RAP expression, and HR < 1 indicates lower estimated risk. Because these pan‐cancer survival screens report nominal *p* values without multiplicity correction, they should be interpreted as hypothesis generating rather than as independent validation of a universal prognostic effect.

### 3.3. Pan‐Cancer Single‐Cell and Spatial Transcriptomic Localization of IL1RAP

**Figure 5:**
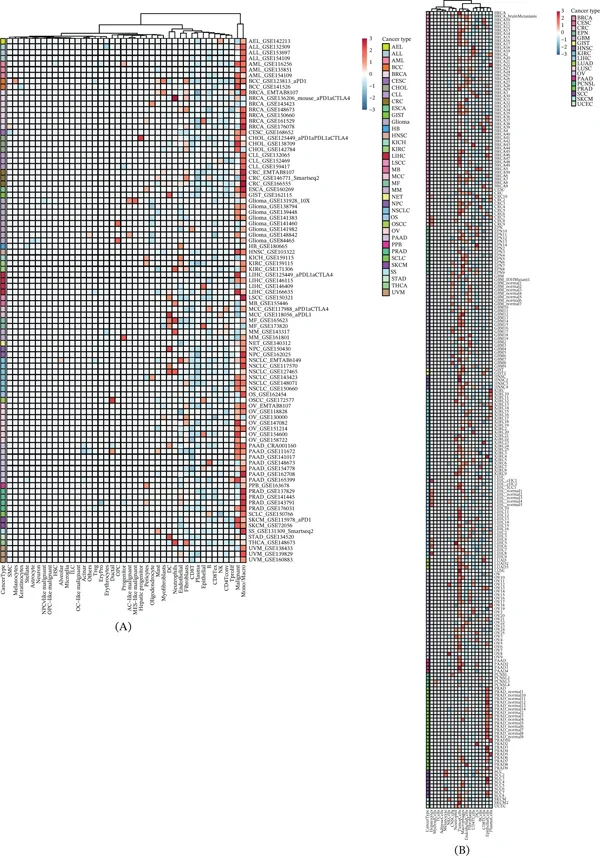
Pan‐cancer single‐cell and spatial characterization of IL1RAP expression. (A) Heatmap of *Z*‐standardized IL1RAP expression across annotated cell populations in the indicated TISCH single‐cell datasets; Euclidean distance and Ward linkage were used for clustering. (B) IL1RAP expression across cell‐type–dominant regions in the indicated 10x Genomics Visium spatial datasets after source‐workflow deconvolution; expression was *Z*‐standardized within the displayed analysis. Sample and section identifiers are shown in the panels. Warmer colors denote higher standardized expression, and cooler colors denote lower standardized expression.

Pan‐cancer single‐cell transcriptomic analysis demonstrated that IL1RAP exhibited clear cell‐type–specific expression heterogeneity rather than uniform expression across cellular compartments (Figure 5A). Across tumor types, IL1RAP expression varied among immune, stromal, and malignant cell populations, indicating that its expression pattern is highly dependent on cellular identity. Hierarchical clustering based on *Z*‐score–normalized expression profiles further showed that cell populations with similar functional characteristics tended to cluster together, suggesting partially conserved IL1RAP expression patterns across cancers. These findings indicate that IL1RAP expression is structured at the cellular level and related to TME composition. Spatial transcriptomic analysis provided complementary evidence supporting spatial heterogeneity of IL1RAP expression within tumor tissues (Figure 5B). IL1RAP expression showed variable distribution across spatial regions, with differences observed among areas enriched for distinct cellular components. The spatial maps indicated that IL1RAP expression is not uniformly distributed but instead varies according to local tissue architecture and cellular composition. These spatial patterns were observed across multiple tissue sections, supporting the robustness of the spatial transcriptomic observations. Together, the integration of single‐cell and spatial transcriptomic analyses indicates that IL1RAP expression is both cell‐type–dependent and spatially heterogeneous within TMEs.

### 3.4. Pan‐Cancer Pathway Enrichment and Functional State Associations of IL1RAP

GSEA showed that IL1RAP expression was associated with multiple Hallmark and KEGG pathways across cancers, with the direction and magnitude of enrichment varying by cancer type (Figure 6A). FDR‐adjusted results are shown for GSEA. CancerSEA‐based functional‐state analysis also identified correlations between IL1RAP expression and proliferative, genome‐maintenance, stress‐response, and immune‐related state scores in selected cancers (Figure 6B). These nominal correlation results indicate covariation with pathway activity and do not establish that IL1RAP regulates the corresponding programs.

Figure 6Pan‐cancer pathway and functional‐state associations of IL1RAP. (A) GSEA of Hallmark and KEGG metabolism gene sets comparing the top 30% versus bottom 30% of IL1RAP expression within each cancer type. Bubble color shows NES direction and magnitude (positive, enrichment in the high‐expression group; negative, enrichment in the low‐expression group), and bubble size represents the displayed FDR metric. Benjamini–Hochberg correction was applied. (B) Pearson correlations between continuous IL1RAP expression and CancerSEA functional‐state scores. Colors identify functional states, and coefficients show correlation direction and magnitude; displayed correlation *p* values are nominal.

**Figure:**
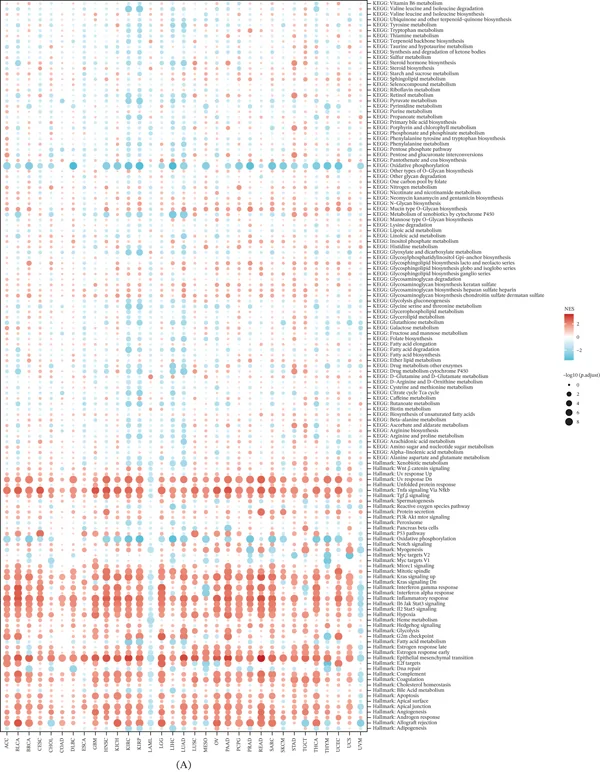
(A)

**Figure:**
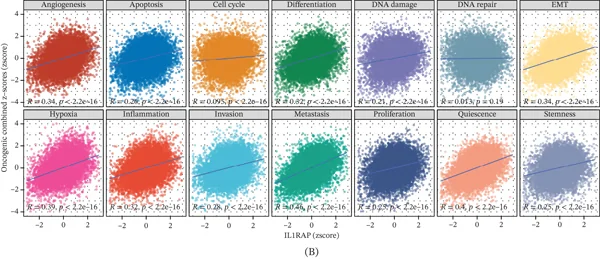
(B)

### 3.5. Pan‐Cancer Immune–Genomic Landscape and Genomic Alteration Features Associated With IL1RAP

Stratification by IL1RAP expression quartile revealed variation in immune‐response and genomic‐state scores across cancer types (Figure 7A). IL1RAP expression also differed among copy‐number categories in several cancers (Figure 7B), and somatic alterations of IL1RAP were generally uncommon and cancer‐type dependent (Figure 7C,D). Spearman correlations with selected genomic features likewise varied across tumors (Figure 7E). These cross‐sectional, nominal associations describe genomic context but do not establish directionality or a causal effect of IL1RAP.

Figure 7Pan‐cancer immune, genomic‐state, and alteration associations of IL1RAP. (A) Row‐standardized mean immune‐response and genomic‐state scores across IL1RAP expression quartiles Q1–Q4. (B) IL1RAP expression across CNV categories; the two‐sided Kruskal–Wallis test was used. (C) Pan‐cancer mutation frequencies for IL1RAP and representative driver genes. (D) IL1RAP somatic‐variant types across cancers. (E) Spearman correlations between continuous IL1RAP expression and selected genomic features. Colors and scales are defined in each panel; positive coefficients indicate concordant increases, and negative coefficients indicate inverse associations. Asterisks denote nominal *p* values ( ^∗^ *p* < 0.05,  ^∗∗^ *p* < 0.01,  ^∗∗∗^ *p* < 0.001,  ^∗∗∗∗^ *p* < 0.0001); no global FDR correction was applied to these displayed associations. Available samples after source‐specific filtering were used, and *n* varies by cancer type and data layer.

**Figure:**
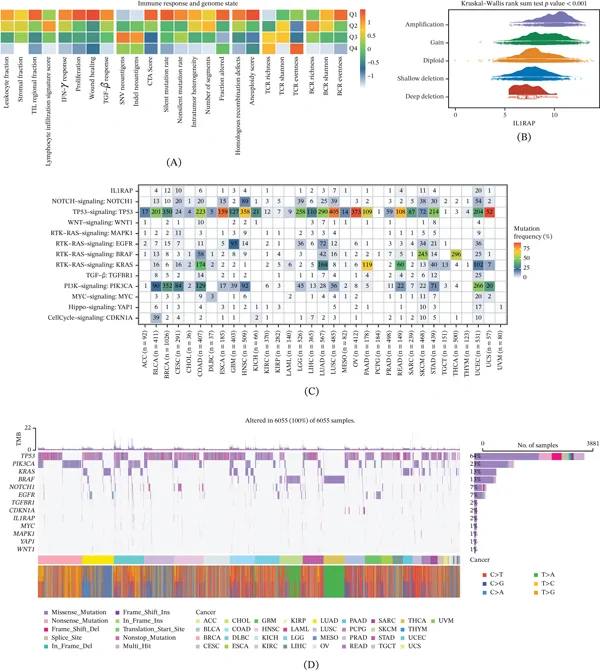
(A)

**Figure:**
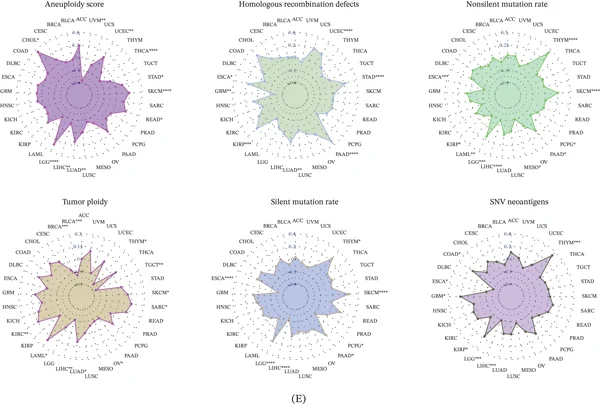
(B)

### 3.6. Pan‐Cancer Associations Between IL1RAP Expression and Pharmacogenomic Drug‐Response Metrics

**Figure 8:**
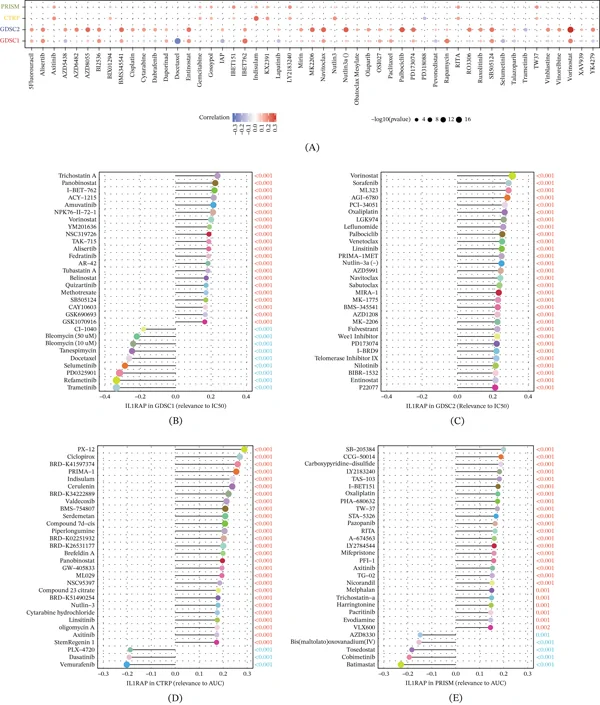
Pan‐cancer correlations between IL1RAP expression and pharmacogenomic drug‐response metrics. (A) Spearman correlations across GDSC1, GDSC2, CTRP, and PRISM. Bubble color denotes correlation direction, and intensity denotes magnitude. (B–E) Top 30 nominally ranked correlations in GDSC1, GDSC2, CTRP, and PRISM. For AUC and IC50, a negative coefficient indicates lower drug‐response values at higher IL1RAP expression (greater apparent sensitivity), whereas a positive coefficient indicates higher values (lower apparent sensitivity). Available cell lines with paired expression and drug‐response measurements were included, so *n* varies by compound and resource. Rankings use nominal two‐sided *p* values without global FDR correction and are hypothesis generating.

Across GDSC1, GDSC2, CTRP, and PRISM, IL1RAP expression showed positive and negative correlations with selected drug‐response metrics (Figure 8A–E). A negative correlation denotes lower AUC or IC50 at higher IL1RAP expression, whereas a positive correlation denotes higher AUC or IC50. The top‐ranked associations differed across resources, consistent with differences in cell‐line composition, assay platform, and response metric. Because compounds were ranked using nominal *p* values and no perturbation experiment was performed, these results are hypothesis generating and do not show that IL1RAP directly determines sensitivity or resistance to any agent.

### 3.7. Association Between IL1RAP Expression and Immune‐Infiltration Patterns in SKCM

In TCGA‐SKCM, CIBERSORT‐based immune‐cell compositions differed descriptively between the median‐defined IL1RAP high‐ and low‐expression groups (Figure 2A). The ssGSEA correlations indicated positive associations with several T‐cell and helper‐cell estimates and negative associations with other immune‐cell estimates (Figure 2B). Representative results from xCell, MCP‐counter, and QUANTISEQ also showed positive or negative correlations with selected microenvironmental components (Figure 2C–F). Because these methods use different reference signatures and modeling assumptions and the displayed *p* values are nominal, convergence in direction across selected estimates should be considered supportive association evidence rather than proof that IL1RAP regulates immune contexture.

### 3.8. Association of IL1RAP With MeTIL Levels and Immunotherapy Outcomes in SKCM

In TCGA‐SKCM, MeTIL scores were higher in the median‐defined IL1RAP‐high group than in the IL1RAP‐low group (Wilcoxon *p* = 0.015; Figure 1A). The online‐generated plot did not provide a standardized effect‐size estimate, so the result is reported as a distributional comparison rather than as a quantified magnitude of effect. OS associations were then examined using cohort‐specific optimal cutoffs in five melanoma immunotherapy datasets (Figure 1B–F). In GSE78220 (anti‐PD‐1), higher IL1RAP was associated with a lower estimated hazard (HR = 0.088, 95% CI 0.014–0.540; log‐rank *p* = 0.0083; high/low *n* = 25/2). In GSE91061 (anti‐PD‐1), higher IL1RAP was associated with a higher estimated hazard (HR = 10.662, 95% CI 1.476–77.037; *p* = 0.019; *n* = 91/10). In GSE106128 (dendritic cell–based vaccination; longitudinal expression profiles), the corresponding association was HR = 3.150 (95% CI 1.415–7.012; *p* = 0.00497; *n* = 27/20). In Nathanson_2017 (CTLA‐4 blockade), the association was inverse (HR = 0.199, 95% CI 0.057–0.697; *p* = 0.0116; *n* = 20/4). In PRJEB23709 (anti‐PD‐1 alone or combined anti‐PD‐1/anti‐CTLA‐4), higher IL1RAP was associated with a higher estimated hazard (HR = 8.021, 95% CI 1.085–59.295; *p* = 0.0414; *n* = 80/11). The opposing directions, wide CIs, imbalanced optimal‐cutoff groups, and heterogeneous treatment/sampling contexts preclude describing IL1RAP as a consistent predictive biomarker. These results instead identify context‐dependent, exploratory survival associations that require independent validation with prespecified cutoffs and standardized patient‐level response data.

## 4. Discussion

Inflammation and immunity are inseparable components of tumor biology, yet they are often analyzed as parallel layers rather than as a coupled system [35]. A recurring challenge in “inflammation–immunity” biomarker studies is that many inflammatory nodes are pleiotropic and cell‐type dependent: The same molecule can reflect tumor‐intrinsic stress adaptation, myeloid remodeling, or lymphocyte activation depending on where it is expressed and how the microenvironment is organized [36, 37]. This is precisely where IL1RAP becomes conceptually interesting. As a signaling adaptor in interleukin‐1 family pathways, IL1RAP represents a plausible integrator of inflammatory cues that can propagate into immune tone, but its utility in cancer is unlikely to be captured by any single dataset or any single analytical layer [38].

A major implication of this work is methodological: An inflammation–immunity association identified in bulk data benefits from triangulation across orthogonal evidence types [39]. Bulk transcriptomes can detect associations but are ambiguous regarding cellular source [40]. Integrating bulk analyses with single‐cell and spatial resources and then examining disease‐anchored clinical datasets can improve interpretability, while still remaining observational [39, 41]. In this context, IL1RAP serves as an example of how an inflammatory signaling component can be evaluated as a microenvironment‐associated marker rather than treated as a generic expression feature.

SKCM is a relevant disease anchor because melanoma outcomes are strongly shaped by immune contexture and immune engagement [42, 43]. In such a setting, biomarkers that reflect immune organization may be more informative than a nonspecific indication of high inflammation [7]. Our analyses suggest that IL1RAP may mark an inflammation–immunity–related state associated with the melanoma immune ecosystem. However, the present data do not establish that IL1RAP itself regulates this ecosystem or predicts benefit from a specific immunotherapy. Any clinical stratification value must be tested against established covariates in independent cohorts using prespecified thresholds [44].

An important conceptual distinction is that IL1RAP should not be interpreted as a universal “good” or “bad” factor across cancers [10]. Inflammatory signaling adaptors often sit upstream of multiple branches that can yield divergent phenotypes across lineages, and expression can be shaped by both genomic regulation (e.g., copy‐number context) and microenvironmental composition [45]. This is a common reason why pan‐cancer studies often produce heterogeneous prognostic directions. Rather than treating such heterogeneity as a weakness, it can be biologically informative: It suggests that IL1RAP is embedded in tumor‐type–specific wiring and that a disease‐anchored interpretation (here, SKCM) is essential before making translational claims. In other words, pan‐cancer provides prioritization, whereas melanoma provides meaning.

The pharmacogenomic layer is explicitly hypothesis generating. Correlations between gene expression and AUC or IC50 can reflect shared cellular programs, lineage, or proliferation rather than direct drug–gene causality [46, 47]. The observed associations may help prioritize compounds for future experimental testing, but cross‐resource differences and the absence of IL1RAP perturbation prevent conclusions about mediated sensitivity or resistance. Accordingly, these data should be viewed as a source of testable hypotheses rather than evidence for treatment selection [48, 49].

The melanoma‐focused findings likewise support evaluation of IL1RAP as a possible stratification marker rather than a standalone response predictor. The current immunotherapy analysis examines OS separation after data‐derived dichotomization; it does not directly compare responders and nonresponders and cannot distinguish predictive from prognostic association. MeTIL provides an orthogonal immune‐related readout, but its association with IL1RAP remains observational and does not prove mechanism [50].

Several limitations should be emphasized. First, the study is retrospective and observational; causal statements about IL1RAP driving immune phenotypes, drug sensitivity, or treatment response are not justified. Second, immune deconvolution and gene set scoring rely on platform‐ and method‐specific assumptions; using multiple algorithms does not eliminate this uncertainty. Third, most pan‐cancer, survival, immune‐infiltration, genomic, and pharmacogenomic screens report nominal *p* values without global multiplicity correction and should be treated as exploratory. Fourth, the melanoma immunotherapy datasets differ in treatment, biospecimen, sampling time, unit of analysis, endpoint annotation, and processing, and no pooled batch correction was appropriate. Fifth, Figure 1 uses cohort‐specific minimum log‐rank *p* cutoffs selected and evaluated in the same datasets, which can inflate apparent separation; the small and imbalanced groups and wide CIs reinforce the need for prespecified validation. Sixth, standardized clinical response categories were not analyzed, so the data do not establish association with response or resistance. Seventh, the MeTIL analysis used the published five‐probe normalized PCA signature, but the current online‐derived output did not provide a standardized effect‐size estimate. Finally, the single‐cell and spatial resources improve localization but do not substitute for melanoma‐specific experimental validation.

Future work should focus on three directions that would materially strengthen the translational narrative: (i) prospective clinical validation in melanoma cohorts with standardized annotations, enabling multivariable modeling and assessment of incremental predictive value; (ii) compartment‐resolved validation, ideally determining which cellular compartments contribute most to IL1RAP‐associated immune phenotypes in melanoma; and (iii) mechanism‐aware perturbation, testing whether modulating IL1RAP‐associated inflammatory signaling reshapes immune organization or therapeutic sensitivity in melanoma‐relevant systems. Importantly, these steps do not require the assumption that IL1RAP is a druggable target; they simply test whether IL1RAP is a reliable state marker and whether the state it marks is actionable.

In summary, this study identifies associations between IL1RAP expression and several inflammation‐, immunity‐, genomic‐, and treatment‐related features in melanoma within a pan‐cancer–informed, multimodal framework. The convergent immune‐related observations support further evaluation of IL1RAP as a state marker, whereas the heterogeneous cohort‐specific survival results and retrospective design require cautious interpretation.

## 5. Conclusion

This integrative pan‐cancer–guided, melanoma‐anchored analysis suggests that IL1RAP may mark an inflammation–immunity–related state in SKCM. Associations with immune‐infiltration estimates and MeTIL scores provide supportive observational evidence, whereas survival associations across melanoma immunotherapy datasets were heterogeneous and based on exploratory optimal cutoffs. IL1RAP should therefore be considered a candidate marker for further mechanistic and prospective validation, not a validated predictor of immunotherapy response or a demonstrated therapeutic target.

## Author Contributions

C.J. and H.L. performed data collection, bioinformatics analyses, and figure generation. C.J. drafted the initial manuscript. J.H. and M.C. conceived and supervised the study. All authors contributed to manuscript writing and critical revision.

## Funding

No funding was received for this manuscript.

## Disclosure

All authors approved the final version of the manuscript.

## Ethics Statement

This study was conducted using publicly available datasets and did not involve direct human or animal participation. Therefore, ethical approval and informed consent were not required.

## Conflicts of Interest

The authors declare no conflicts of interest.

## Data Availability

All datasets analyzed in this study are publicly available. The data can be obtained from The Cancer Genome Atlas (TCGA), the Genotype‐Tissue Expression (GTEx) project, and publicly accessible repositories including GEO and other referenced databases. Additional information is available from the corresponding authors upon reasonable request.
